# Batch Pyrolysis and Co-Pyrolysis of Beet Pulp and Wheat Straw

**DOI:** 10.3390/ma15031230

**Published:** 2022-02-07

**Authors:** Jerzy Chojnacki, Jan Kielar, Leon Kukiełka, Tomáš Najser, Aleksandra Pachuta, Bogusława Berner, Agnieszka Zdanowicz, Jaroslav Frantík, Jan Najser, Václav Peer

**Affiliations:** 1 Faculty of Mechanical Engineering, Koszalin University of Technology, Racławicka Str. 15-17, 75-620 Koszalin, Poland; leon.kukielka@tu.koszalin.pl (L.K.); apachuta@poczta.fm (A.P.); bogusia.berner@wp.pl (B.B.); agnieszka.zdanowicz@s.tu.koszalin.pl (A.Z.); 2 Centre of Energy Utilization of Non-traditional Energy Sources—ENET Centre, VSB—Technical University of Ostrava, 17. listopadu 2172/15, 708 00 Ostrava, Czech Republic; jan.kielar@vsb.cz (J.K.); tomas.najser@vsb.cz (T.N.); jaroslav.frantik@vsb.cz (J.F.); jan.najser@vsb.cz (J.N.); vaclav.peer@vsb.cz (V.P.)

**Keywords:** synergy, generator, biomass, biochar, bio-oil, pyrolysis, gas composition

## Abstract

Granulated beet pulp and wheat straw, first separately and then mixed in a weight ratio of 50/50%, underwent a pyrolysis process in a laboratory batch generator with process temperatures of 400 and 500 °C. The feedstock’s chemical composition and the pyrolysis products’ chemical composition (biochar and pyrolysis gas) were analysed. A synergistic effect was observed in the co-pyrolysis of the combined feedstock, which occurred as an increase the content of the arising gas in relation to the total weight of the products. and as a reduction of bio-oil content. The maximum gas proportion was 21.8% at 500 °C and the minimum between 12.6% and 18.4% for the pyrolysis of individual substrates at 400 °C. The proportions of the gases, including CO, CO_2_, CH_4_, H_2_, and O_2_, present in the resulting synthesis gases were also analysed. The usage of a higher pyrolysis final temperature strongly affected the increase of the CH_4_ and H_2_ concentration and the decrease of CO_2_ and CO concentration in the pyrolysis gas. The highest percentage of hydrogen in the synthesis gas, around 33%_vol_, occurred at 500 °C during co-pyrolysis.

## 1. Introduction

The production of chemical fuels and energy from organic waste facilitates sustainable waste management. Local waste products from the agriculture and agri-food industry are already used for energy purposes. The most common methods for the thermal processing of biomass include incineration and co-incineration. However, the application of these processes is challenging due to the necessity, among others, to use technical solutions that will cope with problems, such as a wide range of chemical composition, shape, and humidity of input materials. It causes difficulties with stabilizing the combustion process and emissions of dusts, hydrogen chloride, furans, dioxins, and ash removal [1,2,3,4,5]. The problems concerning the physical parameters of biomass as a fuel may be solved by its granulation and briquetting [6,7].

Among thermal methods of biomass energy utilization, gasification and pyrolysis can be distinguished [8,9,10]. The chemical transformations occurring during biomass pyrolysis are very complex. The final products are solid residue (biochar), liquid residue (bio-oil), and pyrolysis gas, which is a mixture of gases, primarily flammable ones [11]. Pyrolysis takes place at lower temperatures than combustion. As a result, the subsequent energy recovery from the combustion of pyrolysis gas produces fewer toxic substances in the flue gas than in the case of direct combustion of the fuel [10]. In addition to typical biomass wastes, such as cereal straw and wood, attempts are being made to use all biomass wastes for bioenergy production by pyrolysis, such as, for example, paper mill sludge (PMS), date palm, and spent ground coffee beans [12,13]. Biomass pyrolysis is characterised by variability in the substrate content. The products resulting from this process can be stored and used for energy purposes [14]. The process is influenced by the type of biomass and its pre-treatment as well as reaction atmosphere, temperature, heating rate, and process duration. The amount and composition of the fractions obtained depend mainly on the process temperature [15,16]. The optimum bio-oil and bio-carbon yield are temperatures between 400 and 550 °C. At temperatures higher than 600 °C, bio-oil is converted to gas by secondary cracking. In addition to obtaining the maximum amount of bio-carbon and bio-oil, the target of biomass pyrolysis can also be a hydrogen-rich synthesis gas [15]. Studies of the gas composition produced during pyrolysis have indicated that as the temperature increases, the CO_2_ and CO content decreases, and the H_2_ and CH_4_ content increases [17].

Microwave [18], batch [19], flow, screw, and fluidized bed [20,21] generators can be used to perform biomass pyrolysis. It is experimented with catalytic cracking to refine the pyrolysis products [22]. The preparation of the pyrolysis process requires the supply of an adequate portion of energy in a relatively short time to ensure the required temperature (ca. 300–600 °C on average) [23]. Currently, these are used mainly as external energy sources (natural gas, electricity) to heat the reactor, which means that biomass processing in such a process may not be economically beneficial [24]. In some applications, the pyrolytic gas can be used to heat the reactor [15].

Among the numerous types of waste from agricultural production, straw (the residue from cereal cultivation) and beet pulp play an essential role. Beet pulp is a waste product from beet sugar production. Three main types of waste are generated during beet sugar production: beet pulp, molasses and defecation lime. Defecation lime is produced as a result of introducing CaO or Ca(OH)_2_ into the process in the form of milk of lime. It consists of 12% organic matter by weight, and it contains 0.4% N, 1.1% P_2_O_5_, 0.1% K_2_O, 1.2% MgO, and calcium carbonate in the amount of approximately 30%. Defecation lime, due to its high lime content (CaCO_3_) and the presence of P_2_O_5_, K_2_O, MgO, and Na_2_O, is used as a fertilizer for plant cultivation. Fresh beet pulp contains cellulose and hemicellulose (~40%), pectin (up to 50%), proteins (2%), sugars (2–3%), minerals (2%), and a small number of vitamins and organic acids [24]. Beet pulp can be used as animal feed and as a raw material in cosmetic and paper industries [25]. It can also be used as a raw material for methane production in bioreactors [26,27].

Investigations concerning the beet pulp and straw pyrolysis were carried out using different methods. Most of them were performed using laboratory analytical equipment, where biomass weight micro samples were used as the substrate [28,29,30]. Additionally, using micro samples and applying the Thermogravimetric Analysis (TGA) method, the kinetics of pyrolysis of beet pulp, separated or mixed with lignite, was studied [31,32,33]. Such studies were also carried out in a dual-fluidized bed reactor [21]. The results demonstrated the influence of the final process temperature on the composition of the solid and liquid residues and the gas obtained. As the process temperature increased, the yield of biochar and oil or tar decreased, and the yield of gas products increased [21]. Due to the maximum bio-oil output, the 450 °C value was considered to be the optimum temperature for straw pyrolysis [34]. FTIR spectra (Fourier transform infrared spectroscopy) showed that the process of beet pulp pyrolysis produces not only large amounts of gases, such as CO_2_, CO, H_2_O and CH_4_, and H_2_, but also many volatile aldehydes, ketones, organic acids, and alkanes, and co-pyrolysis involving the pyrolysis of several feedstock materials mixed compared to the pyrolysis of individual feedstock may also produce a synergistic effect concerning some products resulting from the process. This could be an increased yield of bio-oil [35] or an increased proportion of the ash that occurred during the co-pyrolysis of beet pulp with lignite [32]. An increase in the mass proportion of defecation lime in samples with beet pulp resulted in an increase in the mass of biochar from 29.4 to 51.6%. Yet, there was also a decrease in the higher heating value (HHV) of the resulting products, biochar and tar [30,31]. The results showed that the kinetic parameter values were changed with the ratio of beet pulp to lignite. An increase in conversion of mixed raw materials was found with an increase in the weight percentage of beet pulp in the blends. Co-pyrolysis studies of *Miscanthus giganteus* and sugar beet shreds were also performed, examining the adsorption mechanism of alachlor and pentachlorobenzene of chars. The studies demonstrated that biomass conversion temperature affects the production process in terms of chars’ quantity and physicochemical properties [36].

In addition, the pyrolysis process of beet pulp and wheat straw was tried as a method to produce char-based foams, which may find numerous applications, such as absorption, catalysis, and energy storage [37]. The advantage of these products is their high liquid-absorption capacity.

The aim of this study was to evaluate the influence of the composition of the mixture of two types of agricultural waste biomass subjected to the pyrolysis process and to evaluate the influence of the temperature of this process on the quantity of the products obtained in this process, on their composition, and their quantitative relations. The research aimed to search for the influence of the co-pyrolysis of selected raw materials and the final temperature of the process on the percentage relationships of the gases included in the gas obtained in this process. The hydrogen and methane content was of primary concern. The research also covered the search for enhancing the quantity or quality of the products of co-pyrolysis performed on a mixture of feedstock materials compared to the effects of single pyrolysis of these feedstock materials. Beet pulp and wheat straw were selected as feedstock. These wastes of agricultural origin have not been jointly subjected to the pyrolysis process before. It was decided to carry out experiments in the laboratory using a batch generator, which would allow observation of phenomena close to conditions that might occur in similar industrial generators.

## 2. Materials and Methods

### 2.1. Materials

**Figure 1:**
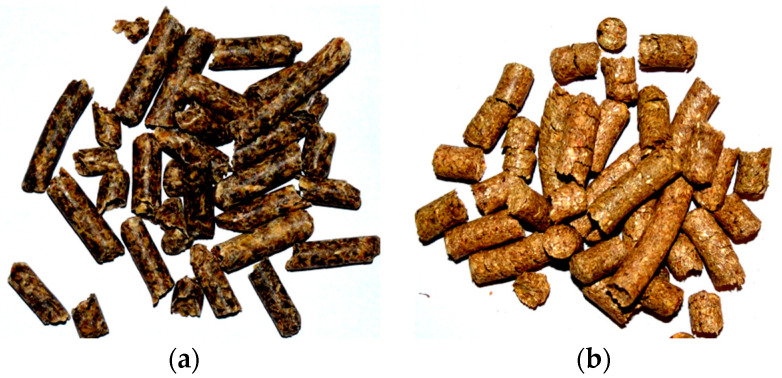
Pellets: (**a**) beet pulp pellets and (**b**) straw pellets.

The primary material used in the study was beet pulp pellets; they were obtained as waste from a sugar factory and wheat straw pellets (Figure 1). The beet pulp pellets used for the study had a diameter of 7 mm and a length of 15–50 mm, while the wheat straw pellets had a diameter of 8 mm and a length in the range of 6–50 mm. The values determined of the density and the bulk density of both feedstock materials are presented in Table 1.

### 2.2. Test Stand

**Figure 2:**
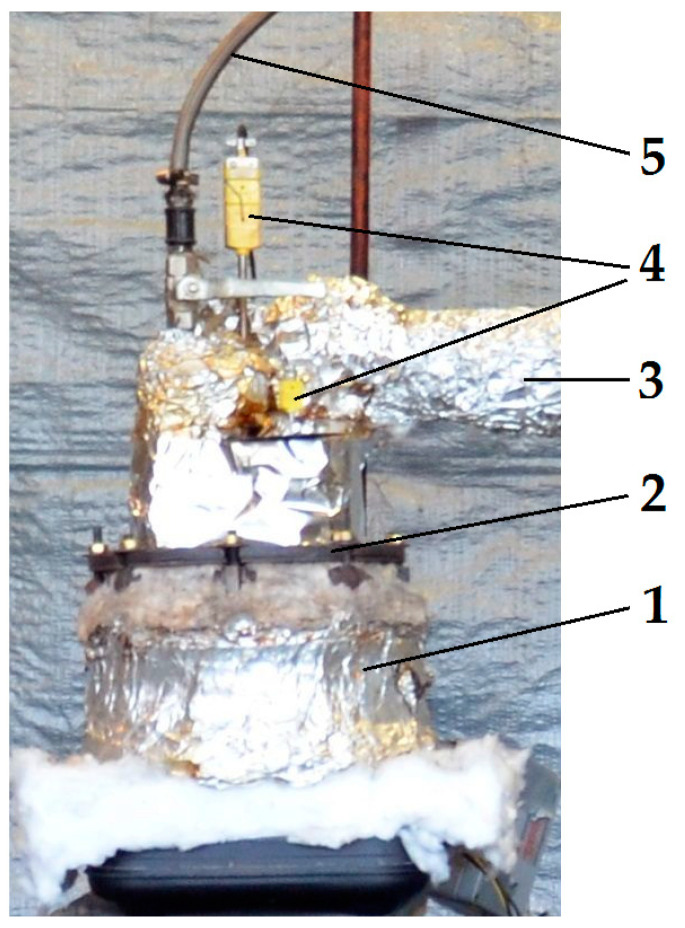
Laboratory pyrolysis reactor: (1) External container, (2) cover, (3) outlet pipe, (4) thermocouples, and (5) inert gas inlet.

The pyrolysis process of the materials was carried out in a laboratory batch reactor, as shown in Figure 2. The reactor was constructed from an outer container covered by a lid into which an inner open container including biomass was inserted. Both containers were made of corrosion-resistant sheet metal. The outer container of the reactor was heated from the outside by two electric heaters. The first heater was a heating element wound on the outer cylindrical wall of the reactor; the second heater was placed under the bottom of the outer container. The amount of the current passing into the external and bottom heating elements was operated independently. In this manner, a better control of the process temperature inside the reactor was achieved. 

The total power of the electric heaters was 930 W. Two thermocouples were mounted in the reactor’s lid to provide information about the current temperature inside the reactor and control this temperature. In addition, in the lid of the reactor, there was a pipe for the discharge of vapours and gases produced as a result of the pyrolysis of the materials, and there was a pipe for an introduction of inert gases into the reactor. The whole reactor was covered with mineral wool lagging to reduce heat loss. A container with biomass was inserted into the reactor. Its size was selected to allow contact with the inner surface of the reactor wall and with its heated base. The volume of the vessel was 2.0 dm^3^. The advantage of using a laboratory batch reactor was, on the one hand, the possibility to carry out more tests over a shorter time period and at a lower cost than in the case of a large commercial device and, on the other hand, to carry out the process on larger weight samples of granulated biomass then it is done in laboratory analytical equipment. 

**Figure 3:**
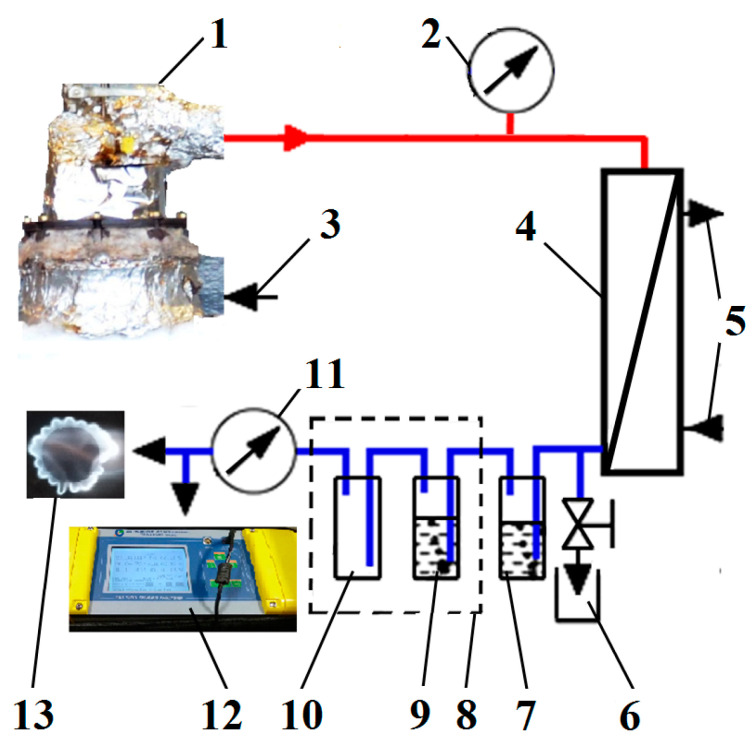
Test stand: (1) Pyrolysis reactor, (2) pressure gauge, (3) electricity power, (4) gas and oil water cooler, (5) inlet and outlet of cool water, (6) container for pyrolysis oil, (7) water scrubber for gas purification, (8) cooling system, (9) impinger in cooling system with isopropyl alcohol, (10) empty impinger in cooling system for cleaning gas, (11) gas flow meter, (12) gas analyser, and (13) burner.

The reactor was connected to a test stand with measuring equipment, which was used to analyse the components of vapours and gases produced during pyrolysis. The complete test stand for analysing the composition of volatile substances from the generator is shown in Figure 3. Due to its high temperature, the gas emitted directly from the reactor contained vapours of substances that are not gases at ambient temperature but are solid or liquid substances, including water and pyrolysis oil, among others. For this purpose, in the gas-flow path, a water cooler for gas products (4), a container for liquefied pyrolysis oil (6), a water scrubber for gas purification (7), and a cooling system (8) were installed. The cooler contained a container with isopropyl alcohol and an empty gas-cooling container. The temperature in the refrigerator was 5 °C.

Prior to starting the experiment, the following vessels were weighed: an empty biomass container, a liquefied pyrolysis oil container, an aqueous gas purification scrubber, an isopropyl alcohol container, and an empty gas-cooling container. Before placing the feedstock into the reactor, the reactor was heated to reach the test temperature, then it was opened, and a weighed portion of the pellets was poured into the biomass container. Once the reactor had been sealed, an inert gas, i.e., nitrogen, was admitted to remove all oxygen from the reactor. As a result of the introduction of the raw fuel and purging with inert gas, the reactor was considerably cooled. While reacting to the cooling, the control system automatically started to reheat the reactor filled with the feedstock material. 

Two pyrolysis temperatures were accepted for each feedstock material: 400 and 500 °C. Each experiment was performed twice. The residence time of the fuel in the reactor depended on the time taken to reach the set temperature and the time that the pyrolysis fluid was released to the vessel. After the completion of the process, the heating was turned off, and the reactor was cooled to 200 °C. This temperature marked the end of the pyrolysis process.

### 2.3. Products of Pyrolysis

The composition of the syngas was analysed with the aid of a Portable Syngas Analyser Gas 3100. The analyser allowed an assessment and recording in time of changes in the volume percentage of gases in the syngas, such as CO, CO_2_, CH_4_, H_2_, and O_2_. In addition, the analyser calculated the LHV (the lower heating value) of the gas based on the composition measured. The total volume of the gas produced during pyrolysis from the feedstock was measured using a PI 0.1 Spectrum laboratory gas flow meter. The flow meter and Portable Syngas Analyser were fed with cooled and purified gas. As the flow rate of the gas produced exceeded the value required by the analyser, the excess was carried off from the system via a separate pipe, and then, it was burnt in burner.

In the composition of the synthesis gas, there were also gases that could not be determined using the sensors installed in the analyser. The percentage content of these gases in the synthesis gas was determined by calculating the difference between 100%_vol_, i.e., the theoretical total volume of all the gas components, and the total volume of the gases determined by the analyser. Unidentified gases in the graphical presentation of the results are denoted by UG letters. Measurements of changes in the synthesis gas composition with the gas analyser were carried out in experiments in which the final temperature of the process was 500 °C.

The weight of the biochar, i.e., the solid residue produced during pyrolysis, was determined once the process had been completed and the reactor opened. For this purpose, the biomass container with the residue was removed and weighed, and then, by subtracting the weight of the empty container, the weight of the solid residue was calculated.

In order to determine the volume of the separated liquid, i.e., pyrolytic oil, the following vessels were weighed again at the end of the process: the container for liquefied pyrolytic oil, the water scrubber for gas purification, the container with isopropyl alcohol and the empty gas-cooling container. In addition, the pipes and the gas and oil cooler (4) were washed with a weighed amount of acetone after cooling. Based on the differences in the weights of the vessels before and after the process and the weight of acetone before and after washing the pipes, the total weight of pyrolytic fluid was determined. Apart from measuring the volume of the gas separated from the material undergoing pyrolysis at a given temperature, its mass was also determined using Formula (1):

Mg = Ms − Mc − Mo(1)

where Mg—mass of pyrolysis gas, g; Ms—mass of feedstock, g; Mc—mass of solid residue after pyrolysis, i.e., biochar, g; Mo—mass of pyrolysis liquid, i.e., bio-oil, g. 

## 3. Results and Discussion

### 3.1. Pyrolysis Products

Prior to testing, an elemental composition analysis was carried out; the combustible substance content and the heat value were established in the feedstock materials. An elemental composition analysis was performed using a LECO CHSN628 analyser. The analysis of the fuel content in the feedstock materials was performed by means of the TGA method, using a TGA 701 analyser. The results of the individual analyses are shown in Table 2.

**Figure 4:**
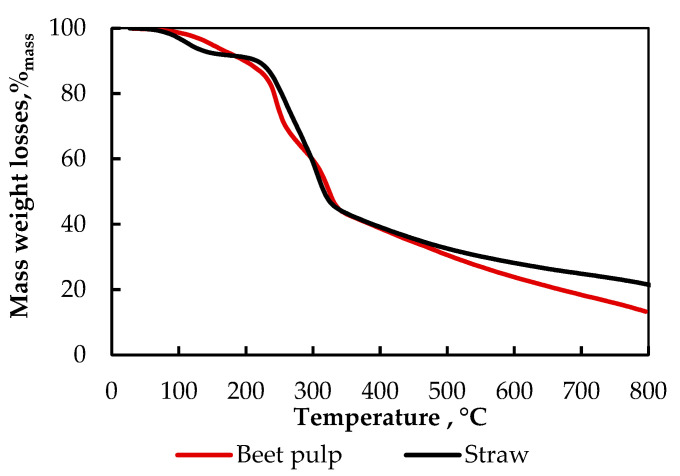
Impact of heating temperature on weight loss of straw and beet pulp pellet samples.

In order to evaluate the changes in the weight of the raw materials used in this study under the influence of temperature, a thermographic analysis of these was carried out. Weight losses were evaluated using the TGA 701 analyser. The results are shown in Figure 4. In the first phase of thermal decomposition (TGA) of biomass, up to about 200 °C, water evaporation was predominant. Between 250 and 320 °C, the decomposition of cellulose, hemicelluloses, and lignin occurred. From 320 to 800 °C, further decomposition of the products was observed.

The analysis demonstrated a high similarity in the weight losses due to annealing in the furnace between the beet pulp and straw samples, which may indicate that their biological composition does not differ too much. 

Beet pulp pellets mixed with wheat straw pellets (both include moisture) at a wet weight ratio of 50/50%_mass_ underwent a co-pyrolysis process in the batch generator. The choice of weight ratio fuels with moisture for the preparation of the mixture was based on the results of the moisture content measurement of each material. The difference in water content between the beet pulp pellet and the wheat straw pellet was 0.2%_mass_. It was concluded that the moisture content of the two materials was comparable. The accepted weight ratios of the pellet mixture determined in relation to wet weight corresponded with high accuracy to the weight ratio to dry weight. In addition to co-pyrolysis, pyrolysis of the individual feedstock materials was performed under the same thermal conditions. Each batch weight of the feedstock material that underwent the process in the reactor was 1100 g. 

The solid residues obtained from the pyrolysis process were also subjected to an ultimate analysis and a combustible matter content analysis. The analyses were carried out in the same way as for the feedstock. The results of the analysis of the solid residue, i.e., biochar, are presented in Table 3. In addition, the hypothetical biochar composition of the raw material mixture, calculated on the basis of biochar composition after pyrolysis of beet pulp and wheat straw individually, was recalculated and presented in Table 3. 

An analysis of the chemical composition of the solid residues indicates an increase in ash content in proportion to the remaining components. This is a natural phenomenon in the case of a thermal emission of organic biomass components. Water previously contained in the raw materials used was mainly transferred to the composition of the post-process fluids, i.e., bio-oil. Comparison of the values of hydrogen-to-carbon and oxygen-to-carbon ratios in the calculated biocarbon composition of a mixture of raw materials with the corresponding values in the biocarbon derived from the co-pyrolysis of these raw materials indicates a more significant reduction in the values of hydrogen-to-carbon and oxygen-to-carbon ratios in the biocarbon derived from the co-pyrolysis than that resulting from the pyrolysis of these materials individually.

In order to evaluate the loss in the composition of the feedstock materials as a result of pyrolysis carried out at 400 and 500 °C, comparative calculations were carried out. For this purpose, it was assumed that there be no change in the ash in biochar content of the ratio to its content in the feedstock materials before pyrolysis. Ash theoretically remained in the solid residue after the process. As the determined the ash content in biochar was much higher than in the raw materials used, the ratio between the ash content in biochar and the ash content in the corresponding feedstock materials was calculated. The results are presented in Table 4. 

Using then the proportions determined, the values of the components in biochar were calculated at the same ash content as in the feedstock material by dividing the values of the other components found in the biochar by the corresponding values in Table 4. Next, the corresponding contents of biochar were subtracted from the content of the components in the feedstock material, and the losses in the content of feedstock material components due to pyrolysis were obtained. The results are presented in Table 5.

The loss of organic matter from the feedstock material ranged from 53.6 to 63.2% of its weight content. The greatest loss of percentage weight content was found for oxygen, carbon, and hydrogen. The slight percentage loss of hydrogen (3.5–4.5%) actually accounted for about 80% of the feedstock material content. Between 400 and 500 °C, the differences in elemental losses were minor. In most comparisons, differences in losses at these temperatures are within of measurement accuracy. 

The impact of the process temperature on the variation of carbon content in solid and liquid residues following pyrolysis was described in studies [15,16]. Similar changes in ash content were also observed in the article [38]. When analysing in a similar manner as this paper the data on ash and carbon content in biochar contained in the study [38], a decrease in carbon loss in biochar can be observed at higher pyrolysis temperatures and assuming that the ash content does not change. 

The values measured for the mass of biochar, pyrolysis fluid, and syngas obtained and the measurement results concerning the volume of the gas obtained from the pyrolysis of biomass at 400 and 500 °C are found in Table 6. The mean values obtained from the measurements and their standard deviations are included. The beet pulp + straw data in Table 6 have been supplemented with calculated average values from the pyrolysis results of the single raw materials. These values are shown next to the experimental data.

A comparison of the values in Table 6, including the importance of their standard deviations, shows that the gas values at 400 and 500 °C obtained during co-pyrolysis are higher than the average gas values obtained from calculations for weight values and volume values also. At 400 and 500 °C, the bio-oil values obtained during co-pyrolysis are lower than the average bio-oil values obtained from the calculation.

For further elaboration of the results, factors were identified that could influence the results obtained. The beet pulp content of the beet pulp + wheat straw mixture was defined as the first factor. According to this assumption, this content accepts the following values: 100%_mass_ when the feedstock material used was beet pulp only, 50%_mass_ when the feedstock material was a mixture of beet pulp and wheat straw, and 0%_mass_ when the feedstock material was wheat straw pellets only. The process temperature and correlations between the beet pulp content in the mixture and the process temperature were accepted as further factors. The raw results obtained were covered by an analysis of variance using Statistica software ver. 13.3 from StatSoftw. The results of ANOVA are presented in Table 7. The significance of the impact of the factors was determined by assessing the value of the *p*-value included, which should not exceed the significance coefficient value of 0.05 when there is a significant impact of the factors.

**Figure 5:**
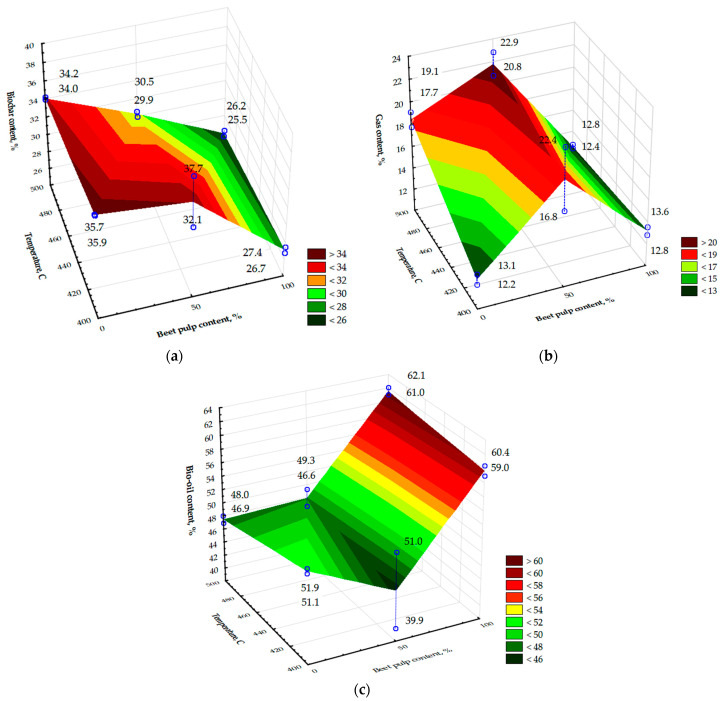
Impact of beet pulp content in the mixture with wheat straw and final process temperature on the weight percentages of pyrolysis products: (**a**) biochar; (**b**) gas; (**c**) bio-oil.

The variance analysis demonstrated the significance of the impact of the beet pulp content in the mixture on all the pyrolysis products values obtained. The variance analysis also demonstrated significance of the impact of the process temperature on the value of the biochar weight obtained. The effect of the process temperature on the weight of the gas secreted out was also close to being significant (*p* = 0.05510). Further analysis of the impact of the composition of the feedstock material and the final temperature of the pyrolysis process on the values of the substances obtained was carried out by analysing the weight percentages of the products, namely solid and fluid residues and gas, concerning the weight of the feedstock material used. The second parameter was the final temperature of the process. The results of the impact of the factors examined on the percentage shares of pyrolysis products were analysed statistically and graphically using the Statistica software, and these are presented as graphs in Figure 5.

The graphs in Figure 5 show and give the values of the pyrolysis product weight percentages calculated from all the test points. The average values of the weight percentages of the pyrolysis products are additionally presented in Table 8. Table 8 also shows the calculated values of the weight shares of the pyrolysis products of beet pulp and straw, determined solely on the basis of data corresponding to the pyrolysis of these separate raw materials.

Based on the results in Table 6 and Table 8 and the graphs presented in Figure 5, it is possible to determine the impact of the beet pulp content in the mixture on the mass proportions of the biochar, bio-oil, and pyrolysis gas. It was observed that an increase the proportion of beet pulp in the substrate decreases the solid residue after the process. The lowest average percentage of biochar, 25.9%, was obtained using 100% beet pulp as feedstock material after pyrolysis was made at a final temperature of 500 °C. However, the significant, unusual influence of beet pulp content on the amount of obtained pyrolysis products was observed at its 50% content in the mixture with wheat straw. This amount of beet pulp in the feedstock resulted in an increase in the amount of gas produced and a decrease in the amount of bio-oil obtained relative to the predicted values as averages calculated from the pyrolysis data of beet pulp and wheat straw separately.

The second factor that impacted the product content was the final temperature of the process, in the range between 400 and 500 °C. Based on Figure 5, there was no considerable effect of temperature on the proportion of biochar. There was an impact of the temperature on the weight percentage of the gas exhaled. An increase in the temperature from 400 to 500 °C resulted in an increase in the percentage of syngas in the total composition of the pyrolysis products. 

It is evident from Table 6 and Table 8 and the graphs in Figure 5 that there was a synergistic effect to the gas content with the feedstock materials mixed, i.e., with 50% beet pulp content and 50% wheat straw content in the substrate mixture. It is evident when comparing the results of the obtained amounts of gas and bio-oil in co-pyrolysis with the calculated average values from the results of the same pyrolysis products of beet pulp and straw—Table 6. The temperature enhanced the synergy, with its growth from 400 to 500 °C strengthening the effect. The content of the resulting gases increased with this composition to a maximum of 21.8% (22.9%—Figure 5). Then, it decreased after pyrolysis of the pellets, either straw alone or beet pulp alone. The highest bio-oil content was found with the highest final process temperature of 500 °C, using pure beet pulp pellets as the feedstock material. Synergy as a result of co-pyrolysis was not only related to the amount of gas but it could also, with 50% beet pulp content and 400 °C temperature, have effected lowering the bio-oil content, similar to another study [35]. No clear symptom of synergy was observed concerning the ash content of the products as described by the authors of the paper [32]. 

The results of the mass and percentage values of the solid residue after pyrolysis in the batch generator are to a small extent similar to the results of the thermogravimetric analysis of beet pulp and straw samples shown in Figure 4. The TGA analysis shows that at temperatures between 400 and 500 °C, there is an overlap of value in the loss of the straw mass compared to the value of loss of beet pulp mass. The results of the batch generator experiments indicate significant differences in the loss of the solid residue mass and differences between the values obtained from the TGA analysis and pyrolysis in the batch generator. It could be due to the larger masses used in the experiments with the generator than in the TGA analysis and the different nature of the phenomena (slower penetration of heat into a much larger volume of biomass and more varied biomass temperature values in the batch generator than in the analyser). Larger particles inside of big mass are penetrated by temperature much more slowly and only to a certain depth compared to single samples that weigh several grams only and that are placed in the analyser oven at the same temperatures. The final products of the co-pyrolysis and pyrolysis and the differences with the results obtained from the TGA analysis may also have been influenced by the chemical reactions of the volatiles (including bio-oil vapour and gas) generated on the wall and the bottom of reactor. The temperature there was higher than inside the reactor. During the passage of these substances through the cooler raw material layer, heat exchange between the volatiles and raw materials can take place as well as a chemical reaction between them.

### 3.2. Pyrolysis Gases

**Figure 6:**
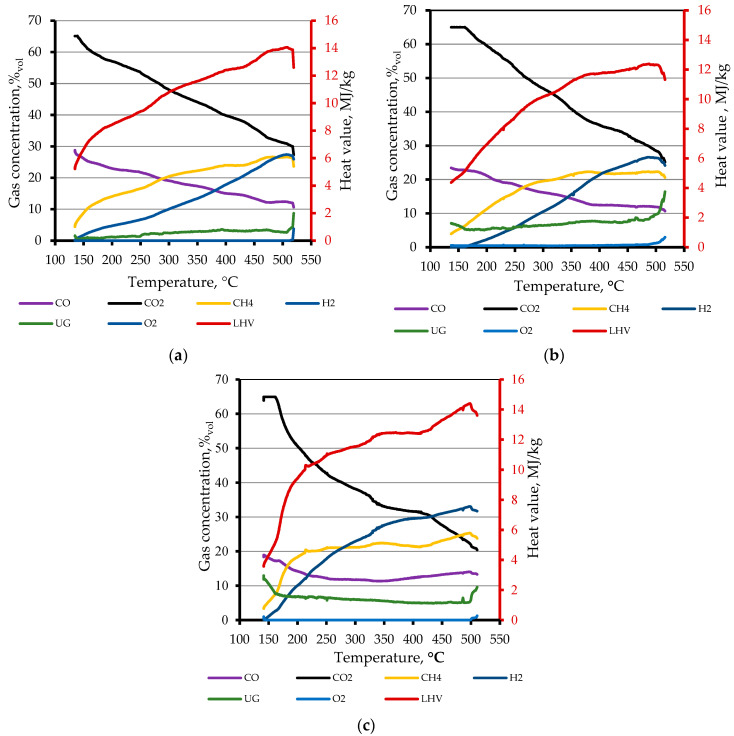
Impact of generator temperature on the volume composition of pyrolysis gas and lower heat value: (**a**) wheat straw; (**b**) beet pulp; (**c**) wheat straw and beet pulp mixture. UG, undefined gases.

Based on the gas composition results recorded with the Syngas Analyzer Gas 3100, graphs of changes in the composition of pyrolysis gases depending on the temperature in the generator were prepared for each substrate used in the experiment. The graphs are presented in Figure 6. In addition to the actual volume content of the identified and unidentified gases, the graphs also show the lower heat value (LHV) course corresponding to the currently identified composition. 

The graph demonstrates that the percentage content of CO_2_, which appeared in the initial phase of the temperature growth (ca. 150 °C), was so high that it exceeded the measuring range of the device (65%). As the temperature in the generator increased, the CO_2_ content decreased almost linearly. Finally, at 500 °C, it reached a value of 30% with the pyrolysis of wheat straw only, a value of 25% when processing mixed feedstock materials, and 20% with the pyrolysis of beet pulp only. The increase in the process temperature also contributed to a decrease in the proportion of carbon monoxide in the gases to 10–12% of the gas volume (500 °C) at all types of feedstock. Despite the decrease in the CO volume, the increase in the temperature increased in the proportion of other energy gases, such as CH_4_ and H_2_. Similar results were obtained by [17,33]. A significant proportion of methane: ca. 10%_vol_, appeared as early as at the temperature range of 150 to 200 °C in the generator. The maximum methane content, 27%_vol_, was obtained during the pyrolysis of wheat straw at a temperature of about 500 °C. The highest proportion of hydrogen in the syngas, about 33%_vol_, was obtained at the process temperature of 500 °C during the pyrolysis of a substrate containing a mixture of beet pulp and wheat straw, which may also be a symptom of a synergistic effect. Since the lower heat value of the syngas depended mainly on the content of CH_4_ and H_2_, while an increase in the proportion of these gases occurred with the growing temperature, the LHV reached the maximum value of 14.1 MJ∙kg^−1^ at 500 °C during the pyrolysis of a mixture of substrates. 

The Syngas Analyzer Gas 3100 was unable to determine the composition of a part of the pyrolysis gas, which is marked in the graphs in Figure 6 as unidentified gases UG. The highest content of these gases appeared as early as approximately 150 °C and at the final temperature of 500 °C in the pyrolysis of beet pulp alone and of the beet pulp and straw mixture, respectively. On the basis of data from articles published concerning the pyrolysis of biomass and beet pulp and straw in particular, it can be assumed that these included carbons, sulphur, and hydrocarbon compounds [18,30,33]. The graph shows that during the whole process, until the final temperature of 500 °C had been reached, the oxygen content in the synthesis gas remained at 0%_vol_. 

Comparing the results obtained from the pyrolysis of beet pulp and wheat straw with the results of previous studies concerning biomass pyrolysis, a similarity can be found in the impact of the process temperature on the composition of synthesis gas. An increase in the temperature caused a decrease in the proportion of biochar as a solid residue. This also caused an increase in the proportion of post-process liquids that occurred in the case of straw and a mixture of straw and beet pulp. The higher-finish temperature of the pyrolysis process influenced the increase in the proportion of gases that occurred in the pyrolysis of beet pulp and the beet pulp and straw mixture [21,28,34].

## 4. Conclusions

The amount of beet pulp in the raw material subjected to pyrolysis influenced the quantity of products. Increasing the content of beet pulp in the mixture and increasing the temperature leads to a decrease in the mass fraction of solid residue (biochar) into pyrolysis products. 

A 50% mass fraction of beet pulp in a mixture with wheat straw caused a synergistic effect consisting of an increase in the mass fraction of syngas in the total of the products compared to the fraction of syngas obtained in the process of pyrolysis of these substrates separately. It was also noted that with 50% beet pulp content in the feedstock and at 400 °C, there was a possibility of a synergy resulting in a decrease in bio-oil content in the products of the process. 

The increasing temperature in the generator impacted the content of individual gases in the pyrolysis gas. It resulted in a decrease in carbon dioxide and carbon monoxide content, causing an increase in hydrogen and methane content. An increase in methane and hydrogen in the syngas substantially impacted a rise in its LHV. 

Due to the occurrence of the synergistic effect and obtaining the highest content of synthesis gas during co-pyrolysis of beet pulp and wheat straw as well as due to obtaining the highest content of methane and hydrogen in the syngas and the highest its LHV, this reaction and its temperature of 500 °C proved to be the optimums for conducted experiments with the pyrolysis of beet pulp and wheat straw.

## Figures and Tables

**Table 1:** Density and bulk density of beet pulp pellets and wheat straw pellets.

Material	Density	Bulk Density
	kg/m^3^	kg/m^3^
Beet pulp	1268.3	660.5
Wheat straw	1201.4	520.6

**Table 2:** Elemental and fuel composition of feedstock materials used in the study.

Raw Materials	Element Concentration, %_mass_							
	C	H	N	S	O	W	A	F
Beet pulp	39.3 ± 1.9	4.5 ± 0.3	0.6 ± 0.2	0.4 ± 0.1	40.5 ± 6.9	11.1 ± 4.8	3.6 ± 0.6	85.3 ± 5.4
Wheat straw	39.4 ± 2.4	5.5 ± 1.0	1.2 ± 0.5	0.6 ± 0.0	31.8 ± 3.6	10.9 ± 1.0	10.6 ± 0.6	78.5 ± 1.6
Beet pulp + wheat straw *	39.35	5.0	0.9	0.5	36.15	11.0	7.1	81.9

* calculated values; W, water; A, ash; F, fuel, organic matter.

**Table 3:** Composition of the solid residue: biochar after pyrolysis process.

Raw Mater.	Process	Ultimate Analysis, %_mass_							Proximate Analysis, %_mass_		
	Temp. °C	C	H	N	S	O	H/C	O/C	W	A	F
Beet pulp	400	59.3 ± 1.5	3.8 ± 0.2	0.9 ± 0.2	0.2 ± 0.0	21.8 ± 2.6	0.064	0.368	0.8 ± 0.1	13.2 ± 3.1	86.0 ± 3.2
	500	61.2 ± 0.4	3.1 ± 0.2	0.9 ± 0.1	0.2 ± 0.0	20.0 ± 0.7	0.051	0.327	0.7 ± 0.1	13.9 ± 1.3	85.4 ± 1.2
Wheat straw	400	49.5 ± 1.1	3.9 ± 0.3	1.1 ± 0.1	0.1 ± 0.0	15.0 ± 0.5	0.079	0.303	0.8 ± 0.0	29.6 ± 0.7	69.6 ± 0.7
	500	51.3 ± 2.1	2.9 ± 0.2	1.3 ± 0.1	0.1 ± 0.0	13.3 ± 0.4	0.057	0.259	0.8 ± 0.1	31.1 ± 1.1	68.9 ± 1.7
Mixture	400	58.2 ± 1.0	2.7 ± 0.0	0.9 ± 0.1	0.1 ± 0.0	17.2 ± 0.5	0.046	0.296	0.7 ± 0.0	20.2 ± 1.6	79.1 ± 1.6
	500	61.1 ± 2.5	1.8 ± 0.2	0.8 ± 0.0	0.1 ± 0.0	12.1 ± 0.2	0.029	0.198	0.7 ± 0.0	23.4 ± 2.6	75.9 ± 2.6
Calculate	400	54.4	3.85	1.0	0.15	18.4	0.071	0.335	0.8	21.4	77.8
mixture	500	56.25	3.0	1.1	0.15	16.65	0.054	0.293	0.75	22.5	77.15

**Table 4:** Ratio between ash content in biochar and ash content in corresponding feedstock materials.

Raw	Temperature, °C	
Materials	400	500
Beet pulp	3.67	3.86
Wheat straw	2.79	2.93
Beet pulp + wheat straw	2.85	3.30

**Table 5:** Loss of individual contents of feedstock material components as a result of pyrolysis.

Raw Mater.	Process Temp. °C	Content Losses during the Process,%_mass_							
		C	H	N	S	O	W	A	F
Beet pulp	400	23.1	3.5	0.4	0.3	34.6	10.9	0.0	61.8
	500	23.4	3.7	0.4	0.3	35.3	10.9	0.0	63.2
Wheat straw	400	21.7	4.1	0.8	0.6	26.4	10.6	0.0	53.6
	500	21.9	4.5	0.8	0.6	27.3	10.6	0.0	55.0
Mixture	400	18.9	4.1	0.6	0.5	30.1	10.8	0.0	54.1
	500	20.8	4.5	0.7	0.5	32.5	10.8	0.0	58.9

**Table 6:** Average weight and volume values of pyrolysis products.

Raw Material	Beet Pulp		Beet Pulp + Straw				Straw	
Temp. °C	400	500	400	400 Calc.	500	500 Calc.	400	500
Biochar, g	297.9 ± 5.2	284.4 ± 5.5	384.2 ± 43.6	346.0	332.6 ± 4.8	329.8	394.1 ± 1.3	375.2 ± 2.1
Bio-Oil, g	656.9 ± 11.1	677.1 ± 8.1	500.2 ± 86.5	611.6	527.3 ± 20.9	599.7	566.3 ± 5.8	522.2 ± 8.4
Gas, g	145.2 ± 5.8	138.5 ± 2.6	215.6 ± 43.0	142.4	240.1 ± 16.1	170.5	139.7 ± 7.0	202.6 ± 10.5
Gas, m^3^	0.110 ± 0.003	0.103 ± 0.004	0.155 ± 0.016	0.116	0.226 ± 0.071	0.129	0.122 ± 0.010	0.154 ± 0.010

**Table 7:** Results of variance analysis.

		Biochar, g				Bio-Oil, g			
Factors	Degree of Freedom	SS	MS	F	*p*	SS	MS	F	*p*
Beet pulp cont.	2	18,600.0	9300.0	4310.0	0.00000	52671	26335	19.200	0.00246
Temperature,	1	2347.0	2347.0	7.095	0.03734	3.5193	3.5193	0.0030	0.96122
Beet p. cont. + Temp.	2	851.0	425.0	1.286	0.34287	3080.4	1540.2	1.1250	0.38478
		Gas, g				Gas, dm^3^			
Factors	Degree of freedom	SS	MS	F	*p*	SS	MS	F	*p*
Beet pulp cont.	2	15,309.0	7654.5	19.92	0.00224	1.4362	0.71811	7.7320	0.02184
Temperature,	1	2168.9	2168.9	5.644	0.05510	0.3105	0.31053	3.3435	0.11723
Beet p. cont. + Temp.	2	2435.6	1217.8	3.169	0.11502	0.2979	0.14897	1.6040	0.27666

**Table 8:** Average values of weight percentages of pyrolysis products.

Raw Material	Sugar Beet		Sugar Beet + Straw				Straw	
Temp. °C	400	500	400	400 Calc.	500	500 Calc.	400	500
Biochar, %_mass_	27.1	25.9	34.9	31.5	30.2	30.0	35.8	34.1
Bio-Oil, %_mass_	59.7	61.6	45.5	55.6	47.9	54.6	51.5	47.5
Gas, %_mass_	13.2	12.6	19.6	13.0	21.8	15.5	12.7	18.4

## Data Availability

The data presented in this study are available on request from the corresponding author.
